# Association between education and television viewing among older working and retired people: a comparative study of Finland and Japan

**DOI:** 10.1186/s12889-018-5860-4

**Published:** 2018-07-25

**Authors:** Taishi Tsuji, Airi Amemiya, Kokoro Shirai, Sari Stenholm, Jaana Pentti, Tuula Oksanen, Jussi Vahtera, Katsunori Kondo

**Affiliations:** 1 Center for Preventive Medical Sciences, Chiba University, 1-8-1 Inohana, Chuo Ward, Chiba City, Chiba, Japan; 2 Department of Health Education and Health Sociology, School of Public Health, The University of Tokyo, 7-3-1 Hongo, Bunkyo Ward, Tokyo, Japan; 3 Department of Public Health, Graduate School of Medicine, Osaka University, 2-2 Yamadaoka, Suita City, Osaka, Japan; 4 Department of Public Health, University of Turku and Turku University Hospital, 20014 Turun yliopisto, Turku, Finland; 5 Finnish Institute of Occupational Health, Topeliuksenkatu 41 b (Headquarters), 00250 Helsinki, Finland; 6 Department of Gerontology and Evaluation Study, Center for Gerontology and Social Science, National Center for Geriatrics and Gerontology, 7-430 Morioka-cho, Obu City, Aichi Japan

**Keywords:** Television watching, Sedentariness, Elderly, Work status, Retirement

## Abstract

**Background:**

Educational attainment is associated with physical activity among older people. However, little is known about its association with sedentary lifestyle in European as well as Asian nations. This study aims to examine the associations between educational attainment and daily television viewing as an indicator of a sedentary lifestyle among older working and retired people in Finland and Japan.

**Methods:**

We used cross-sectional harmonized data from two cohorts, the Finnish Public Sector study (*n* = 10,744) and the Japan Gerontological Evaluation Study (*n* = 2493), evaluating individuals aged 65–75 years old. We defined high-duration television viewing as ≥4 h per day. Poisson regression was used to examine the association between educational attainment and high-duration television viewing, stratified by the current working status. Models were adjusted for age, sex, household size, smoking, alcohol, body mass index, chronic diseases, mental disorders, and physical activity.

**Results:**

Of the participants, 27% in Finland and 30% in Japan reported high-duration television viewing. Compared with a low education (≤9 years), Finnish and Japanese retirees with a high education (≥13 years) had less high-duration television viewing [prevalence ratio, PR 0.68 (95% confidence interval 0.63–0.73) and 0.66 (0.55–0.79), respectively]. The corresponding PRs for Finnish and Japanese retirees with intermediate education were also lowered [0.89 (0.83–0.95) and 0.79 (0.68–0.91), respectively]. Among older people still at work, educational attainment was associated with high-duration television viewing among the Japanese but not among the Finnish.

**Conclusion:**

A similar association between educational attainment and high-duration television viewing in Finland and Japan particularly after retirement suggests a robust and consistent impact of educational attainment on a sedentary lifestyle after retirements.

**Electronic supplementary material:**

The online version of this article (10.1186/s12889-018-5860-4) contains supplementary material, which is available to authorized users.

## Background

Longer duration of television (TV) viewing, a correlate of a sedentary lifestyle, is associated with an increased risk of adverse health outcomes, such as obesity [1–3], diabetes [3, 4], cardiovascular disease [5, 6], cancer [7, 8], and mortality [6, 9]. The potential mechanisms for the adverse relationship between TV viewing and health outcomes include reduced energy expenditure compared with other sedentary activities, such as reading and driving a car, and an increased food and total energy intake while viewing TV [3]. Compared with younger or middle-aged people, the duration of TV viewing tends to increase with age in older people, particularly after retirement [10]. The daily TV viewing duration has been found to be approximately 50 min longer for people aged 65–74 years compared with those aged 55–64 years and was approximately 2 h longer for unemployed people compared with full-time workers of this age [11]. This further suggests that this behavioral change poses an additional threat to health of older people. Therefore, high-duration TV viewing could be used as a screening tool to identify susceptible groups for timely interventions.

Several studies have shown that older people with a high socioeconomic status (SES) have a higher level of leisure-time physical activity (PA) and exercise more than the older people with a lower SES [12, 13]. To measure the SES of older people, educational attainment, which is normally fixed earlier in life than occupational class or income, is often regarded as a first-choice indicator because problems of reverse causation are much less serious [14, 15]. Murakami and colleagues reported that older individuals who have a higher educational attainment are more likely to have higher levels of PA [12]. A sedentary lifestyle, however, is not exclusive from leisure-time PA and may have adverse effects on health outcomes independently from leisure-time PA [3, 16]. Therefore, the determinants of PA and sedentary lifestyle are not necessarily the same. Although some previous studies have reported that a low SES may have an adverse effect on a sedentary lifestyle among youth [17] and adults (partially including older people) [18–20], there is little evidence of the social determinants of a sedentary lifestyle focusing on older people irrespective of nationality or cultural background [21, 22]. Therefore, this study aimed to examine the association between educational attainment and sedentary lifestyle evaluated by the high duration of TV viewing among older working and retired people. Furthermore, we investigated the association in both Finland, one of the nations with the highest level of welfare, and Japan, the nation with the world’s highest proportion of older people, to clarify whether a universal relationship exists between two nations’ older populations with different races and cultural backgrounds.

## Methods

### Sample

We collected data from surveys conducted among older people in Finland and Japan. The Finnish data were derived from the Finnish Public Sector (FPS) cohort study. FPS study participants were employees representing a wide range of occupations working in 10 towns and six hospital districts. The data used in this study was restricted to participants aged 65–75 who responded to the 2013 survey inquiring information on duration of TV viewing [*n* = 10,744, response rate = 65% (24,679/38,230)].

The Japanese data were obtained from the Japan Gerontological Evaluation Study (JAGES), a population-based, cross-sectional study of individuals aged 65 years or older without disability from 31 municipalities, which was conducted between August 2010 and January 2012 (*n* = 112,123; response rate = 66.3%) [23]. The data used in the present study were from a sub-sample of the JAGES participants residing in the cities of Kashiwa, Nagoya, and Kobe, of which one-fifth provided information on the duration of TV viewing (*n* = 5360). Participants aged 76 years or older (*n* = 2037) were excluded to harmonize with the FPS samples’ age range. In addition, we excluded 556 participants whose status of TV viewing, educational attainment, or current working status were unknown, and 274 participants never had a job. The studies included a total of 2493 (1336 men and 1157 women) participants from Japan and 10,744 (2199 men and 8545 women) participants from Finland.

### Measurement of the duration of TV viewing

In the FPS study, the duration of daily TV viewing was assessed with the following question: “For how many hours do you sit while watching TV/video every day?” In the JAGES, the duration was assessed with the following question: “How much television (including videos) do you watch in a day on average?” Response options were < 1 h, 1–2 h, 2–3 h, 3–4 h, and ≥ 4 h in both questions. We categorized ≥4 h into “high-duration” of TV viewing on the basis that the threshold elevated the risk of all-cause and cardiovascular disease mortality [24].

### Measurement of educational attainment

Educational attainment was categorized into three levels: (i) ≤9 years, i.e., comprehensive school (Finland) and junior high school (Japan); (ii) 10–12 years, i.e., post-compulsory secondary general academic and vocational education (Finland) and high school and technical college (Japan); and (iii) ≥13 years, i.e., university degree.

### Current working status

In the FPS study, a survey response to a question “What is your current working status?” was used. Participants who answered “working full-time” or “part-time” were classified as workers (*n* = 699), and those who answered “retired” (*n* = 10,010) or “not working from other causes” (*n* = 35) were classified as retired. In the JAGES, the participants were asked “What is your current working status?” Those who answered “having a paid job” were classified as workers (*n* = 739), and those who answered “retired” were classified as retired (*n* = 1754). Those who answered “never had a job” were excluded from the analysis (*n* = 274).

### Covariates

Other covariates were age (continuous), sex, household size (1, 2, or ≥ 3 individuals), smoking (never, former, or current smoker), alcohol consumption (non-drinker or current drinker), chronic diseases (having one or more of the following diseases: cancer, cardiovascular diseases, stroke, and diabetes mellitus), mental disorders (a score of ≥4 in the 12-Item General Health Questionnaire [25] in the FPS study and a score of ≥11 in the Geriatric Depression Scale [26, 27] in the JAGES), and moderate/vigorous PA (< 1 day per week or ≥ 1 day per week). In the FPS study, the participants were asked about their average weekly hours of leisure-time PA (including commuting) within the previous year spent walking, brisk walking, jogging, and running, or their equivalent activities. The participants were classified based on whether they performed brisk walking (and equivalent) or a higher intensity of PA for 1 h per week or longer. In the JAGES, the participants were asked about their frequency of low-, middle-, and high-intensity PA and were classified based on whether middle- and/or high-intensity PA was practiced at least once a week or less than once a week. Body mass index (BMI) was calculated as the self-reported weight divided by the square of the self-reported height and then categorized as < 18.5, 18.5–24.9, 25–29.9, and ≥ 30. Missing values were treated as dummy variables.

### Statistical analysis

A Poisson regression analysis stratified by the current working status was performed to examine the effect of educational attainment on the duration of TV viewing. Model 1 was adjusted for age and sex. Model 2 was adjusted for smoking, alcohol consumption, BMI, household size, chronic diseases, and mental disorders. Model 3 was further adjusted for moderate/vigorous PA. Prevalence ratios (PRs) and their 95% confidence intervals (95% CIs) were calculated. We also conducted sensitivity analyses with outcomes set to cut points of ≥3 h and ≥ 2 h per day for high-duration TV viewing. Statistical analysis was performed using the SAS statistical software, version 9.1.3 (SAS Institute, Inc., Cary, North Carolina) and IBM SPSS Statistics 22 (IBM Corp. Armonk, New York).

## Results

Table 1 shows the descriptive cohort-specific data of the participants combined and by their working status. In the Japanese study, 46% were women compared with 80% in the Finnish study. The proportion of moderate/vigorous PA one day per week or more was higher in Finland than in Japan. The prevalence of high-duration TV viewing was somewhat lower in Finland (27%) than in Japan (30%). Retired participants had a high-duration TV viewing more frequently than those still working in both countries (Finland: 28% vs. 16%; Japan: 34% vs. 19%).

**Table 1:** Descriptive data of participants for each cohort and each current working status

	Finland						Japan					
	Total		Working		Retired		Total		Working		Retired	
	(*n* = 10,744)		(*n* = 699)		(*n* = 10,045)		(*n* = 2,493)		(*n* = 739)		(*n* = 1,754)	
Daily TV viewing (n, %)												
<2 hours	1,309	12.2%	156	22.3%	1,153	11.5%	455	18.3%	183	24.8%	272	15.5%
2-<3 hours	3,148	29.3%	261	37.3%	2,887	28.7%	627	25.2%	221	29.9%	406	23.1%
3-<4 hours	3,392	31.6%	173	24.7%	3,219	32.0%	672	27.0%	198	26.8%	474	27.0%
≥4 hours	2,895	26.9%	109	15.6%	2,786	27.7%	739	29.6%	137	18.5%	602	34.3%
Education (n, %)												
Low: ≤9 years	1,958	18.2%	65	9.3%	1,893	18.8%	683	27.4%	217	29.4%	466	26.6%
Middle: 10–12 years	3,362	31.3%	180	25.8%	3,182	31.7%	1,031	41.4%	276	37.3%	755	43.0%
High: ≥13 years	5,424	50.5%	454	64.9%	4,970	49.5%	779	31.2%	246	33.3%	533	30.4%
Age (mean, SD)												
Years	68.5	2.6	67.4	2.3	68.5	2.7	70.1	2.8	69.5	2.7	70.4	2.8
Sex (n, %)												
Men	2,199	20.5%	197	28.2%	2,002	19.9%	1,336	53.6%	457	61.8%	879	50.1%
Women	8,545	79.5%	502	71.8%	8,043	80.1%	1,157	46.4%	282	38.2%	875	49.9%
Household size (n, %)												
1 person	3,590	33.4%	278	39.8%	3,312	33.0%	371	14.9%	102	13.8%	269	15.3%
2 people	6,385	59.4%	372	53.2%	6,013	59.9%	1,260	50.5%	345	46.7%	915	52.2%
≥3 people	244	2.3%	27	3.9%	217	2.2%	763	30.6%	263	35.6%	500	28.5%
Missing	525	4.9%	22	3.1%	503	5.0%	99	4.0%	29	3.9%	70	4.0%
Body mass index (n, %)												
<18.5 kg/m^2^	99	0.9%	3	0.4%	96	1.0%	149	6.0%	36	4.9%	113	6.4%
18.5–24.9 kg/m^2^	4,071	37.9%	272	38.9%	3,799	37.8%	1,805	72.4%	534	72.3%	1,271	72.5%
25.0–29.9 kg/m^2^	4,091	38.1%	291	41.6%	3,800	37.8%	454	18.2%	148	20.0%	306	17.4%
≥30.0 kg/m^2^	1,949	18.1%	109	15.6%	1,840	18.3%	44	1.8%	14	1.9%	30	1.7%
Missing	534	5.0%	24	3.4%	510	5.1%	41	1.6%	7	0.9%	34	1.9%
Smoking (n, %)												
Never	7,986	74.3%	510	73.0%	7,476	74.4%	1,155	46.3%	328	44.4%	827	47.1%
Former	2,022	18.8%	133	19.0%	1,889	18.8%	802	32.2%	251	34.0%	551	31.4%
Current	694	6.5%	55	7.9%	639	6.4%	338	13.6%	109	14.7%	229	13.1%
Missing	42	0.4%	1	0.1%	41	0.4%	198	7.9%	51	6.9%	147	8.4%
Alcohol (n, %)												
Non-drinker	2,265	21.1%	128	18.3%	2,137	21.3%	1,195	47.9%	316	42.8%	879	50.1%
Drinker	8,452	78.7%	569	81.4%	7,883	78.5%	1,129	45.3%	380	51.4%	749	42.7%
Missing	27	0.3%	2	0.3%	25	0.2%	169	6.8%	43	5.8%	126	7.2%
Current chronic disease (n, %)												
No	7,397	68.8%	523	74.8%	6,874	68.4%	1,215	48.7%	363	49.1%	852	48.6%
Yes	3,266	30.4%	174	24.9%	3,092	30.8%	590	23.7%	148	20.0%	442	25.2%
Missing	81	0.8%	2	0.3%	79	0.8%	688	27.6%	228	30.9%	460	26.2%
Common mental disorders (n, %)												
No	9,451	88.0%	656	93.8%	8,795	87.6%	1,966	78.9%	594	80.4%	1,372	78.2%
Yes	1,203	11.2%	39	5.6%	1,164	11.6%	81	3.2%	18	2.4%	63	3.6%
Missing	90	0.8%	4	0.6%	86	0.9%	446	17.9%	127	17.2%	319	18.2%
Moderate/vigorous physical activity (n, %)												
<1 day/week	3,174	29.5%	173	24.7%	3,001	29.9%	1,031	41.4%	365	49.4%	666	38.0%
≥1 day/week	7,484	69.7%	520	74.4%	6,964	69.3%	1,310	52.5%	338	45.7%	972	55.4%
Missing	86	0.8%	6	0.9%	80	0.8%	152	6.1%	36	4.9%	116	6.6%

Table 2 shows the association between educational attainment and high-duration TV viewing by the working status for each cohort. In both cohorts, irrespective of the working status, the level of education was inversely associated with unadjusted values for high-duration TV viewing. After adjusting for all covariates, the associations attenuated but remained significant with one exception, the FPS participants were still working. The fully adjusted prevalence compared with those with a low education was 0.68-fold (95% CI: 0.63–0.73) for high and 0.89-fold (0.83–0.95) for intermediate education among retired FPS cohort members. The corresponding PRs for retired JAGES cohort members were 0.66 (0.55–0.79) and 0.79 (0.68–0.91), respectively. Among working JAGES cohort members with high education compared with low education, the prevalence of high-duration TV time was almost the same than that among the retirees, in which the fully adjusted prevalence was 0.63-fold (0.42–0.93). An additional table presents the results of the sensitivity analyses with outcomes set to TV viewing durations of ≥3 h and ≥ 2 h per day (see Additional file 1). A similar, albeit marginally weaker tendency of the relationship was observed for these lower cut points for high-duration TV viewing.

**Table 2:** Association between educational attainment and high-duration of TV viewing for each cohort and each current working status

	Crude model		Model 1		Model 2		Model 3	
	PR	95% CI	PR	95% CI	PR	95% CI	PR	95% CI
Finland								
Working (*n =* 699)								
Low: ≤9 years	1.00	ref.	1.00	ref.	1.00	ref.	1.00	ref.
Middle: 10–12 years	0.87	(0.51 – 1.47)	0.90	(0.52 – 1.54)	0.93	(0.55 – 1.55)	0.96	(0.56 – 1.62)
High: ≥13 years	0.55	(0.33 – 0.92)	0.59	(0.36 – 0.98)	0.77	(0.47 – 1.26)	0.78	(0.47 – 1.29)
Retired (*n =* 10,045)								
Low: ≤9 years	1.00	ref.	1.00	ref.	1.00	ref.	1.00	ref.
Middle: 10–12 years	0.82	(0.76 – 0.88)	0.83	(0.77 – 0.90)	0.89	(0.82 – 0.95)	0.89	(0.83 – 0.95)
High: ≥13 years	0.54	(0.50 – 0.58)	0.54	(0.50 – 0.59)	0.67	(0.62 – 0.72)	0.68	(0.63 – 0.73)
Japan								
Working (n = 739)								
Low: ≤9 years	1.00	ref.	1.00	ref.	1.00	ref.	1.00	ref.
Middle: 10–12 years	0.77	(0.54 – 1.09)	0.74	(0.53 – 1.05)	0.73	(0.51 – 1.03)	0.75	(0.53 – 1.06)
High: ≥13 years	0.62	(0.42 – 0.92)	0.62	(0.42 – 0.92)	0.61	(0.41 – 0.91)	0.63	(0.42 – 0.93)
Retired (*n =* 1754)								
Low: ≤9 years	1.00	ref.	1.00	ref.	1.00	ref.	1.00	ref.
Middle: 10–12 years	0.76	(0.66 – 0.88)	0.75	(0.65 – 0.87)	0.77	(0.67 – 0.89)	0.79	(0.68 – 0.91)
High: ≥13 years	0.63	(0.53 – 0.74)	0.61	(0.51 – 0.73)	0.64	(0.54 – 0.77)	0.66	(0.55 – 0.79)

PR: prevalence ratio, CI: confidence interval

Model 1: crude model + age and sex

Model 2: model 1 + household size, smoking, alcohol, body mass index, current chronic disease, and common mental disorders

Model 3: model 2 + moderate/vigorous physical activity

## Discussion

The present study found that educational attainment is similarly associated with the duration of TV viewing among older people after retirement in Finland and Japan, which suggests the robust and consistent positive impact of higher educational attainment on avoiding a sedentary lifestyle among those who have retired from their job. This association was found to exist independently of moderate/vigorous PA. Furthermore, the association was also significant among the older current workers in Japan; however, it was not significant among that subgroup in Finland.

Our results regarding retired populations are in line with those of earlier reports indicating a significant association between high education and habitual exercise in older people [12]. Even if the assumed confounders and mediators were adjusted in the present study, the strength of the association between educational attainment and duration of daily TV viewing hardly changed among those older people who no longer work. Income, factual knowledge, enduring cognitive or emotional skills, and social network are regarded as mechanisms that link education to health behaviors and health outcomes [28]. Another possibility is that individuals with a higher education have a good pension which enables them to engage in various leisure-time activities, which require expenditure of money rather than TV viewing, an option less likely for those with a low education and smaller pension. Thus, a high duration of TV viewing would be among the pathways maintaining inequalities in health among older people.

Among older people still working, educational attainment was associated with high-duration TV viewing only in Japan, whereas among retirees, a similar inverse association was observed in both countries. Thus, cultural backgrounds such as those between Finland and Japan, may lead to different pathways through which education is linked to behavior-related risks when people are still at work. According to a survey comparing 10 European countries regarding how one spends time in everyday life, Finnish people had the longest reading time, whereas their TV viewing was moderate [29]. Conversely, as observed from the results of this study, the Japanese taste for TV viewing may be stronger than that of Finnish people. Therefore, in Japan, even older workers tend to spend their indoor leisure time on TV viewing, particularly if they have a low educational attainment. Because TV viewing further reduces individuals’ energy expenditure compared with, e.g., reading as food and total energy intake is increased due to a tendency to eat while watching TV [3], there is a need for preventive measures to shorten the TV viewing especially among the older people with a low educational attainment in both countries.

A strength of this study is that it is based on harmonized variables from two large cohorts of the older people: one from Finland, a Scandinavian welfare state, and one from Japan, one of the world’s highest longevity nation. However, this study also has several limitations. First, a sedentary lifestyle was indirectly evaluated by TV viewing time (including videos) based on self-reported questionnaires in the present studies. Other screen-time behaviors (e.g., computer and video games) and overall sitting time were not considered. Second, the question formats in each cohort differed for potential mediators, particularly moderate/vigorous PA. However, as further adjustment for PA had little effect on the observed associations, such heterogeneity is an unlikely source of major bias in the comparisons. Third, we used educational attainment as a measure of SES in both studies. A more comprehensive measurement of SES at an old age would also include income and occupational status, given that educational attainment is a determinant of future income and occupation [28]. Fourth, because we did not collect compatible variables of environmental factors between two cohorts, we only focused on individual factors. Previous studies have, however, reported that, for example, neighborhood poor walkability was associated with higher durations of daily TV viewing in adults [21, 30]. Further investigation considering built environment factors are required. Finally, the data were cross-sectional. Although the time order between educational attainment and TV-viewing time at an old age is likely to be correct, the exposure and potential mediators were measured simultaneously.

## Conclusion

Lower educational attainment was associated with high-duration TV viewing, which is similar in Finland and Japan, particularly after retirement. This suggests a robust and consistent impact of educational attainment on a sedentary lifestyle after retirement.

## Additional file

Additional file 1: **Table S3.** Association between educational attainment and TV viewing durations (≥3 h and ≥ 2 h per day) for each cohort and each current working status. The additional table presents the results of the sensitivity analyses with outcomes set to TV viewing durations of ≥3 h and ≥ 2 h per day. (XLSX 16 kb)

## Abbreviations

BMI: Body mass index
CI: Confidence interval
FPS: The Finnish Public Sector
JAGES: the Japan Gerontological Evaluation Study
PA: Physical activity
PR: Prevalence ratio
SES: Socioeconomic status
TV: Television
